# Zinc against COVID-19? Symptom surveillance and deficiency risk groups

**DOI:** 10.1371/journal.pntd.0008895

**Published:** 2021-01-04

**Authors:** Marcin P. Joachimiak

**Affiliations:** Environmental Genomics and Systems Biology Division, Lawrence Berkeley National Laboratory, Berkeley, CA, United States of America; The University of Hong Kong, HONG KONG

## Abstract

A wide variety of symptoms is associated with Severe Acute Respiratory Syndrome Coronavirus 2 (SARS-CoV-2) infection, and these symptoms can overlap with other conditions and diseases. Knowing the distribution of symptoms across diseases and individuals can support clinical actions on timelines shorter than those for drug and vaccine development. Here, we focus on zinc deficiency symptoms, symptom overlap with other conditions, as well as zinc effects on immune health and mechanistic zinc deficiency risk groups. There are well-studied beneficial effects of zinc on the immune system including a decreased susceptibility to and improved clinical outcomes for infectious pathogens including multiple viruses. Zinc is also an anti-inflammatory and anti-oxidative stress agent, relevant to some severe Coronavirus Disease 2019 (COVID-19) symptoms. Unfortunately, zinc deficiency is common worldwide and not exclusive to the developing world. Lifestyle choices and preexisting conditions alone can result in zinc deficiency, and we compile zinc risk groups based on a review of the literature. It is also important to distinguish chronic zinc deficiency from deficiency acquired upon viral infection and immune response and their different supplementation strategies. Zinc is being considered as prophylactic or adjunct therapy for COVID-19, with 12 clinical trials underway, highlighting the relevance of this trace element for global pandemics. Using the example of zinc, we show that there is a critical need for a deeper understanding of essential trace elements in human health, and the resulting deficiency symptoms and their overlap with other conditions. This knowledge will directly support human immune health for decreasing susceptibility, shortening illness duration, and preventing progression to severe cases in the current and future pandemics.

## Introduction

At the moment, the pandemic caused by Severe Acute Respiratory Syndrome Coronavirus 2 (SARS-CoV-2) is wreaking havoc worldwide. This is already the third instance of a coronavirus originating from a zoonotic reservoir and crossing into humans in the 21st century [1,2]. In fact, after the SARS epidemic in 2003/2004, researchers predicted further coronavirus epidemics as early as 2007 [3]. With an increasing world population and progressive urbanization, this is expected to continue. Multiple reports detailing the incidence and fatalities of the Coronavirus Disease (COVID-19) by age group indicate that older patients and those with preexisting underlying conditions are most at risk [4–9]. Newer results are showing that approximately half of infected individuals are asymptomatic [10–12]. It is likely that many of these “asymptomatic” individuals present with subtle, transient, or symptoms not considered to be hallmarks of COVID-19 by the research community or the affected individual. In general, the human phenotype and disease symptom landscape is complex, including multifactorial effects of genetics and the environment, as well as differing severity and subfeatures of phenotypes. However, even if COVID-19 symptoms overlap or are masked by other conditions such as stress and insomnia, the large number of cases worldwide provides a rich dataset and opportunity for learning.

The SARS-CoV-2 virus has presented us with a major challenge to find effective ways to mitigate and ultimately end this pandemic. Epidemiological features, such as the relatively high transmission rates (*R*0 = approximately 2–3 [13,14]), the high frequency of asymptomatic cases [10,11], many different symptomatic presentations with unknown biomarkers and progression [15], and prolonged viral shedding in convalescents [16], all uniquely complicate this viral outbreak. A deficit of research on vaccines, infectious diseases, and their mechanisms, in particular for viral diseases, and a general lack of international and national collaborative frameworks for biomedical data and knowledge exchange have been contributing factors to slow the progress against COVID-19. Similarly, technological, social, and political difficulties have resulted in the limited availability, timeliness, and performance of reverse transcription PCR (RT-PCR) tests for presence of virus, serological tests for viral antigens, and antibody tests for host immune response. Given these challenges, one of the hopes is that individuals at higher risk of infection or progressing to severe cases can be identified early to improve healthcare management and clinical outcomes. Information on symptom grouping, overlaps, and progression can be leveraged to find associations and build models explaining even subsets of cases. Such knowledge and models can support symptom surveillance, triage for interventions, and predictions of patient outcomes in a more realistic manner, for example, considering symptom overlaps and potential mechanisms. Here, we consider COVID-19 and zinc deficiency, with their related symptoms as an example, and show that knowledge about symptom overlap and underlying mechanisms provides a needed path for enhanced risk mitigation and diagnosis during a pandemic.

### COVID-19 symptoms versus human phenotypes related to zinc deficiency

Reports from healthcare workers on the front lines have identified a variety of human phenotypes that appear to be associated with SARS-CoV-2 infection, with new symptoms being recognized over time [17]. Symptoms of severe cases of COVID-19 appear to be better characterized and more consistent, accounting for roughly 20% of cases [18,19]. On the other hand, symptoms for milder cases appear to show greater variation, as expected for the larger number of individuals who present as a mild or “asymptomatic” COVID-19 disease [18,19]. These data are still being collected and reviewed. However, there are multiple mobile symptom tracking efforts that should help standardize the collection and deployment of this important knowledge, even when it is being reported directly by patients or healthcare workers (e.g., https://covid.joinzoe.com/us, https://www.apple.com/covid19/, https://coronavirus.health.ok.gov/symptom-tracker, and https://intermountainhealthcare.org/covid19-coronavirus/covid19-symptom-checker/). We believe that many COVID-19 cases described as “asymptomatic” may be harboring subtle and/or transient symptoms, some of which may be difficult for an individual to notice or report. In other cases, they may also present with noticeable symptoms that overlap or are masked by other conditions. For example, among reported COVID-19 symptoms, there is a growing number of anecdotal reports of asymptomatic or mild cases associated with a decrease or loss of sense of smell and/or taste [20–25]. Recently, it was confirmed that 87% of mild to moderate COVID-19 cases exhibited a loss of smell [26]. These symptoms correspond to the human phenotypes of hyposmia (reduced ability to smell), anosmia (loss of ability smell), dysgeusia (distortion of taste), and ageusia (loss of taste). These sensory symptoms have been known to occur post-viral infection and are usually associated with viral infection of the nasal passages and sinusitis [22]. With over 51 million infected individuals as of November 10, 2020 [27], congestion is one of the many possible COVID-19 symptoms [28]. Recently, it has been shown that sensory neurons are not a likely target for SARS-CoV-2 due to lack of angiotensin-converting enzyme 2 (ACE2) expression [29]. Nevertheless, ACE2 is expressed in cells that provide metabolic and structural support to olfactory sensory neurons and in certain populations of stem cells and blood vessel cells [29]. This finding suggests a potential mechanism for the disruption of olfactory signaling in COVID-19 via neuron-supporting cells.

Symptoms can overlap between different human conditions or diseases and individuals can present with different symptoms for the same condition or disease. COVID-19 appears especially challenging in this regard due to the large variety of symptoms and organs involved [15,30]. Dedicated funding and research efforts are needed so we can learn these distributions of symptoms and their context across the landscape of diseases and individual patient cases. The new COVID-19 mobile symptom tracking apps are one example fitting into this effort, and another are newly established public consortia aiming to harmonize data across institutions [31]. Data and resulting knowledge from these efforts will help to identify actionable information in support of disease surveillance, diagnosis, and clinical outcome management.

One example of a rich collection of human symptoms collected across populations and time are those related to specific nutritional deficiencies. An important focus for COVID-19 is immune activity and health, which is often linked to zinc [32,33]. There are a number of zinc deficiency symptoms, including developmental growth retardation, hypogonadism, cognitive impairment [34], loss of appetite, impaired immune function, and also potentially hair loss, diarrhea, impotence, eye and skin lesions, delayed healing of wounds, the aforementioned taste abnormalities, and mental lethargy [35]. Many of these symptoms overlap with symptoms known to occur during viral infections as well as other diseases.

An intriguing set of overlapping symptoms has to do with loss of taste and smell perception. Gustatory dysfunction is most commonly associated with allergic rhinitis, chronic rhinosinusitis, and upper respiratory infection [36,37], the latter two involving infections and all three involving inflammatory immune response. There have also been multiple reports linking the loss of taste or smell to zinc deficiency, either due to chemotherapy and metal chelating agents [38–40], or due to nutritional deficiencies, especially in older populations [41,42], also caused by medical procedures such as dialysis [43]. As a result, zinc has been proposed in the treatment of taste disorders [44,45]; however, zinc supplementation for chemotherapy-induced loss of smell or taste, which is often transient, has not led to improvements in this condition [38,39].

We know that cellular inflammation causes cellular loss of zinc [46,47] and conversely that zinc deficiency leads to increased inflammation [48]. However, the mechanism by which zinc deficiency causes loss of smell or taste is unknown, making it difficult to design effective therapies. While it may be unlikely that COVID-19–related changes in smell or taste perception are related to zinc deficiency, recent data suggest a possible indirect link through reduced odorant receptor levels in response to innate immune signaling [49]. Intriguingly, it has been proposed that olfactory receptor neurons may initiate rapid immune responses at early stages of disease [50], which would be consistent with functional coevolution of human immune response with viral disease vectors associated with early detection at viral entry points into the body. Combined with known effects of inflammation leading to loss of cellular zinc, a testable hypothesis emerges regarding a sustained and massive immune response resulting in depletion of zinc levels. For COVID-19 specifically, this is further supported by symptoms known to enhance risk for zinc deficiency including sweating, loss of appetite, vomiting, diarrhea, inflammation, and increased metabolic demands due to oxygen deficiency. One problem in COVID-19 research, and more broadly emerging diseases, is the large number of proposed hypotheses with varying support and lacking clear and accessible avenues for validation. The potential loss of olfactory perception via zinc deficiency can be directly tested by monitoring zinc levels and tracking sensory perception improvements in response to supplementation. This would allow to rule out spurious symptom overlaps and with the additional benefit of identifying cases of zinc deficiency during a critical period for optimal immune health.

### Zinc is essential for human immune function and activity against infectious pathogens

Zinc is an essential trace element for all kingdoms of life [51]. It is even involved as a structural ligand in recently solved SARS-CoV-2 crystal structures including for proteins considered as primary drug targets: the main protease [52,53]; RNA-dependent RNA polymerase [54]; the papain-like protease [55]; nonstructural protein 10 (NSP10) [56]; and the complex of NSP12, NSP7, and NSP8 [57]. After iron, zinc is the second most common trace element found in the human body. It plays multiple roles in human biology, predicted to be involved in >10% of human proteins [58], including special roles in the immune system. The importance of zinc for the immune system has been reviewed elsewhere [32–34,59–71]. Briefly, zinc is important for the skin’s barrier functionality, for gene regulation in lymphocytes, and for normal development and function of cells mediating nonspecific immunity such as neutrophils and natural killer cells [60]. The broad impact of zinc on key features of human immunology is based on the panoply of roles for zinc, including in basic cellular functions such as DNA replication, RNA transcription, cell division, and cell activation, and related to potentiation of apoptosis, as well as antioxidant and membrane stabilizing properties. Perhaps most relevant to COVID-19, severe zinc deficiency causes lymphopenia and increased apoptosis of lymphocytes [72], effectively leading to a form of immunodeficiency. Additionally related to symptoms of severe COVID-19, zinc is a known anti-inflammatory agent and decreases production of inflammatory cytokines and oxidative stress biomarkers [73]. There is also evidence that zinc levels are involved in a negative feedback loop controlling immune response via nuclear factor kappa B (NF-κB), implicating zinc deficiency in sepsis and excessive inflammation [74]. Nevertheless, we know relatively little about the hierarchical importance and interdependencies of trace elements for human health and disease, warranting more studies and enhanced data collection.

### COVID-19: Immune health and zinc deficiency risk groups

How might this be relevant to the novel SARS-CoV-2 virus, a human pathogen to which we are exposed for the first time in history? A recent review focused on the role of zinc supplementation for COVID-19, mostly regarding promising direct antiviral effects which unfortunately still lack clinical data [61]. Others have mentioned zinc along with other supplementation as an adjunct therapy to support immune health [62,75–82]. Even in mild COVID-19 cases, our immune response is acting to recognize, neutralize, and clear viral particles, and with increasing disease severity, these responses are intensified or perhaps short-circuited [83]. For severe COVID-19 cases, this may eventually lead to Acute Respiratory Distress Syndrome (ARDS) amid serious side effects caused by the body’s own immune system. However, even for mild cases, there are reports of the infection occurring in cycles where people feel alternately better and then worse [84], likely due to cycles of viral replication, estimated to be about 10 hours [44], and possibly also due to waves of host immune response. Furthermore, SARS-CoV-2 infection appears to have a prolonged incubation period and disease course even for mild cases [85], elevating the importance of interventions which can shorten infectivity and duration. As mentioned earlier, COVID-19 symptoms include multiple additional risks for zinc deficiency, which may exacerbate existing risk groups. Elderly [63] and nutritionally challenged populations are known to have higher rates of zinc deficiency [86], which has been associated with poor outcomes for pneumonia [87,88]. Interestingly, over-supplementing zinc can also cause loss of smell or taste, apparently associated with nasal applications of zinc (now discontinued [89–93]). Assuming no zinc deficiency, the daily Food and Drug Administration (FDA) tolerable upper limit (TUL) dose of zinc is <40 mg, while the Recommended Daily Allowance (RDA) is 8 mg for women and 11 mg for men, for persons 18 years or older [35]. However, many available supplements have multiple times the RDA amount [94], while others do not contain this essential trace element at all.

Zinc deficiency is known to impact immune function resulting in an increased susceptibility to infections [32,33]. Some aspects of zinc supplementation are well studied, although studies of effects on the duration of the common cold have been inconclusive, perhaps because of improper dosing, timing, or delivery route [95]. Multiple human trials have shown conflicting evidence for zinc supplementation improving outcomes in patients with pneumonia. In some cases, zinc supplementation shortened the duration of viral infections and improved outcomes in children with lower respiratory infections [60,96–98] but not in others [99], and even in some cases for elderly patients with pneumonia [100] but not in others [52]. In addition to undernourished children and the elderly, vegan and vegetarian populations are particularly susceptible to zinc deficiency, as plant-based diets will contain more phytates which are known to interfere with zinc bioabsorption [44,45,101,102]. As a result, vegetarians are recommended to increase their RDA amount by 50% [103] and use techniques for increasing zinc bioavailability such as soaking and sprouting grains and seeds [104,105]. In addition, the main dietary sources of zinc are certain seafood (oysters (673%), crab (49%), lobster (31%)), beef (64%), and chicken (22%), with only beans (26%) and pumpkin seeds (20%) providing strict vegetarians with significant amounts (percentages are FDA daily value per serving) [35]. This may also suggest that people who eat more meat and seafood are less likely to suffer from zinc deficiency and may potentially have more robust immune systems, consistent with some reports of immune system problems in vegetarians [106,107]. However, phytates are common in cereal grains; thus, zinc malabsorption effects are likely to extend across a wide range of diets [102]. These dietary effects may be masked or enhanced by genotype and local environmental effects, but should be considered in multifactorial analysis of COVID-19 susceptibility and clinical outcomes.

Unfortunately, zinc deficiency is common in the developing world, with over 2 billion affected individuals [108]. It is less well known that more moderate zinc deficiencies are observed in many regions worldwide [86]. Therefore, minimally, in the case of the elderly, immunocompromised, or undernourished populations, existing data supports the hypothesis that zinc supplementation during the SARS-CoV-2 pandemic would be a safe, inexpensive, and adjunct treatment to reduce the risk of infection and severe disease progression. We highlight populations and groups at risk of zinc deficiency in Table 1 and note that these risk factors may frequently compound, e.g., nutritional deficiency combined with preexisting conditions or environmental exposure. Not all zinc deficiency mechanisms are known, and we list what are considered to be known causes. Some multivitamins supplements lack zinc because the flavor of zinc is considered unpleasant [104]; thus, even people who regularly take supplements may have insufficient zinc levels.

**Table 1:** Zinc deficiency risk groups and associated mechanisms.

Category	Group	Mechanism
Age	Elderly	Reduced dietary intake and impaired absorption [109,110], epigenetics [48]
Age	Children	Mainly nutritional deficiencies in the developing world and vulnerable populations
Lifestyle	Vegetarian	Low consumption of zinc-rich foods, higher phytates in diet (zinc chelation) [111]
Lifestyle	Vegan	Low consumption of zinc-rich foods, higher phytates in diet (zinc chelation) [111]
Lifestyle	Diet high in cereal grains	higher phytates in diet (zinc chelation) [111]
Lifestyle	High alcohol intake	Increased excretion [112] and malabsorption [112]
Lifestyle	Supplementation failure	Zinc not always included in multivitamins
Infectious diseases	GI tract conditions (e.g., diarrhea or vomiting due to infection)	E.g., malabsorption, increased excretion, and reduced dietary intake
Other diseases	Many*	E.g., malabsorption, increased excretion, oxidative stress, and genetic variants in proteins binding zinc or involved in zinc biology
Conditions	Many**	E.g., malabsorption, increased excretion, membrane permeability, and oxidative stress
Medical procedures	Radiation treatment, bariatric surgery, dialysis, hemodialysis	E.g., oxidative stress [113], reduced intake, and malabsorption
Drug treatments	Many***	E.g., oxidative stress and zinc chelation
Exposure	Mercury, tartrazine	E.g., possible ROS and membrane disturbances [114]

* E.g., Acrodermatitis enteropathica [115], Wilson disease [116], sickle cell disease [117], atopic dermatitis [118], anorexia nervosa [119], pancreatic insufficiency [120], gestational diabetes [121], chronic kidney disease [122], chronic liver disease [123], prediabetes [124], and small bowel/GI diseases like IBS [125].

**E.g., Pregnancy [126], exclusively breastfed older infants [127], hyperactivity [128], exercise-induced increase in gut permeability [129].

***E.g., Quinolone and tetracycline antibiotics [35], diuretics [35], sodium valproate [130], ACE inhibitors [131], exclusive parenteral nutrition [132], and chemotherapy [133].

ACE, angiotensin-converting enzyme; GI, gastrointestinal; IBS, inflammatory bowel disease; ROS, reactive oxygen scavenger.

There also exist dependencies, which are not fully understood, with other essential nutrients, such as vitamin D3, which is thought to regulate homeostasis of some trace elements including zinc [134]. A recent study also showed benefits of vitamin D supplementation for suppression of COVID-19 cytokine storm [135]. However, newer research is revealing that it may be severe immunosuppression and not cytokine storm that is the characteristic of COVID-19 [136], leading to immune enhancement as a focus of therapy. As more studies are performed and hypothesis tested, we may learn whether such potentially conflicting results are due to missing context or misled hypothesis. To this end, nutritional deficiency risk groups with their associated mechanisms or features can aid in studies and analysis by providing a source of categorized factors. Such factors can be linked to clinical and epidemiological data and thus enhance the granularity and explanatory power of statistical analysis or machine learning results.

### Strategies for zinc supplementation and monitoring in COVID-19

While zinc is relatively nontoxic, its excess can cause malabsorption of other elements (e.g., copper [137]). In addition, there are a number of known drug interactions with zinc, the most severe being with two HIV inhibitors raltegravir and elvitegravir, along with 32 more moderate interactions [138]. Excess zinc is excreted, hence the continual need for dietary and supplementary sources including supplementing daily with repeated smaller doses. These are some crucial considerations to determine and monitor proper zinc supplementation regimens. Thus, rational nutritional supplementation should take into account dynamic changes such as those due to symptoms associated with enhanced risk for specific deficiencies (e.g., sweating or diarrhea leading to increased loss of zinc), tuned to the expected context for a specific disease such as COVID-19.

While we may not know the optimal zinc supplementation for an individual in the context of COVID-19, supplementation in the context of nutritional deficiency has been demonstrated to offer many positive outcomes for the immune system and specifically the ability to resist infectious pathogens [60, 139–146]. However, it is important to distinguish between chronic zinc deficiency in contrast to zinc deficiency acquired upon viral infection, since different monitoring and supplementation strategies are required. The former requires prophylactic therapy to correct a nutritional deficiency and improve immune health, likely resulting in lower infection rates and less severe disease progression. The latter is an adjunct therapy, used to maintain immune health during viral infection, and requiring careful monitoring and dynamic interventions in response to, for example, episodes of symptoms known to lead to zinc deficiency (e.g., sweating or diarrhea).

Unfortunately, current zinc clinical measurements do not accurately reflect intracellular zinc levels [147]. Monitoring zinc levels is complicated by the fact that zinc is distributed as a cofactor across a wide range of macromolecules [148]. However, even if there is no accepted biomarker for zinc deficiency [149], zinc levels can be easily, if not necessarily very accurately, monitored using blood serum, washed scalp hair, urine, saliva, and fingernails [150]. Performing multiple zinc assay types should be considered to improve accuracy. Even if these measurements do not reflect well the intracellular concentration, they provide a relative measure of zinc levels and thus can help assess supplementation effects. The question of optimal and nontoxic levels of zinc will need to be addressed on a per patient basis. Going forward, nutritional status and supplementation effects of key nutrients should be considered a vital component of all personalized health initiatives.

### Current status of zinc supplementation and therapies for COVID-19

Despite the unprecedented speed of research and clinical trials for new antiviral drugs and symptom treatments for COVID-19, which are also occurring in parallel with vaccine development efforts, the timeframe for being able to produce results with confidence may unfortunately still be measured in years. As of August 28, 2020, there are at least 12 ongoing or proposed clinical trials in the United States for COVID-19 that involve zinc as either a preventative or combination therapy [151]. There is an intriguing mechanistic hypothesis that providing zinc along with zinc ionophores as antiviral therapy may lead to a combined beneficial effect [152–156]. There has been one case study report describing beneficial effects of zinc supplementation for COVID-19 progression [157], and more recently, the first in vivo evidence of zinc supplementation for better COVID-19 outcomes [158]. It has also been reported that COVID-19 patients had significantly lower zinc levels compared with healthy individuals, and this was associated with a greater than 5-fold increased likelihood of developing complications [159]. Since supplementation strategies will be different for preexisting zinc deficiency versus a deficiency acquired during SARS-CoV-2 infection, with acute deficiency during viral infection requiring active monitoring and interventions, it is important to distinguish these cases and their combination.

### Accelerating literature analysis: Toward automating nutritional deficiency risk tables

The rate of published research related to COVID-19 has been at the unprecedented level of thousands of papers per week [160]. This is in addition to the already available biomedical literature on, for example, related viruses. Moreover, many aspects of human health are complex and multifactorial with interdependencies between genetics, environment, nutrition, life course, and lifestyle, to name a few factors. Given this volume of knowledge and expected complexities, future efforts will need to make use of computational approaches such as natural language processing (NLP) to provide more machine-friendly compilations of knowledge for public health. These methods encompass automatic concept recognition from literature supported by existing ontologies and vocabularies [161], as well as discovery of similar and related literature concepts by learning vector representations [161,162]. The application of these tools would allow to more efficiently create updatable nutritional deficiency risk tables outlining specific susceptible groups and potential mechanisms, across a wide range of important molecules important for human nutrition. This research direction can contribute to harnessing the available knowledge in literature to better expose complex biological relationships, such as between trace elements, nutritional deficiencies, and aspects of immune health. An effective organization of our existing scientific knowledge, and in particular capabilities of linking to new data, would be a significant advance for combating pandemics caused by a novel species.

## Conclusions

Current estimates are that 40% to 70% individuals worldwide will become infected over the course of this pandemic in the absence of strong mitigation efforts [163]. We believe that there is a critical need for research and guidance on enhancing baseline human health and specifically the function of the human immune system for individuals worldwide. So far, there have only been limited reports of providing immunity-enhancing supplementation and neither included zinc: one was for National Health Service (NHS) healthcare workers [164] and another for COVID-19 patients over 50 years of age in Singapore [165]. Based on an extensive review of the existing literature presented here, zinc should be included as part of preventative supplementation for COVID-19 and in general for support of immune health. Even given the limited clinical data on zinc as an adjunct therapy for COVID, based on its known safety and limited drug interactions, zinc supplementation should also be considered in the context of zinc deficiency acquired during a viral infection and host immune response.

Healthy individuals with a robust immune system have clearly a better starting point for the difficult COVID-19 viral infection, with expected positive effects on clinical outcomes such as shortening the duration of even just the sub-severe cases. This, in parallel with other efforts and interventions, is likely to decrease the number of severe COVID-19 cases overall, due to a more robust immune response in the population. This is especially critical for vulnerable populations, also in developed countries [166], where safe and cheap interventions are desperately needed. Our limited testing capability for virus presence and the resulting symptom triage for performing a test (e.g. fever, shortness of breath) can potentially delay treatment and increase the number of severe cases. Therefore, actions focused on understanding and improving the human immune system are a critical step to help mitigate negative outcomes. At this point in the pandemic, an important goal is shortening the illness duration and decreasing the risk of severe disease, to alleviate pressures on healthcare systems and ultimately achieve widespread immunity to SARS-CoV-2 in the human population. These strategies will be highly relevant for future emerging viral and other pathogens.

## Methods

Literature searches were performed using the PubMed, Google Scholar, and COVIDscholar [167] literature search engines. For zinc in the context of COVID, we based literature search results on COVIDscholar, which returned 241 articles, documents, and clinical trials for zinc related to COVID-19 (September 17, 2020). We supplemented these searches with PubMed and Google Scholar (September 17, 2020). Clinical trials for COVID-19 involving zinc were obtained from the National Institutes of Health (NIH) clinical trials site [168]. For informing specific sections of the manuscript and construction of Table 1, the following queries were performed: “zinc deficiency,” zinc AND nutrition, zinc AND aging, zinc AND immune, zinc AND antiviral, zinc AND virus, zinc AND infection, zinc AND pneumonia, zinc AND “drug interactions.” All searches were performed between July 2 and November 9, 2020. Query results were curated by selecting the most recent publications and removing redundancy, as well as articles with fewer citations or from lower impact journals.

**Key Learning Points**

- Zinc is the second most abundant essential trace element in the human body with critical roles in immune health and response to infectious diseases.
- Zinc deficiency is common even in the developed world and risks for deficiency can compound.
- Overlap between symptoms in different conditions, for example, a nutritional deficiency versus Coronavirus Disease 2019 (COVID-19), can be used to suggest clinical tests, diagnosis, and triage interventions.
- Zinc provides a safe and cheap alternative to enhance immunity worldwide, both to correct chronic nutritional deficiencies and to address acute deficiencies resulting from a viral infection and host immune response.

**Top Five Papers**

1. Heyneman CA. Zinc deficiency and taste disorders. Ann Pharmacother. 1996;30:186–187.
2. Wessels I, Maywald M, Rink L. Zinc as a gatekeeper of immune function. Nutrients. 2017;9.
3. Baum MK, Lai S, Sales S, Page JB, Campa A. Randomized, controlled clinical trial of zinc supplementation to prevent immunological failure in HIV-infected adults. Clin Infect. Dis. 2010;50:1653–1660.
4. Carlucci PM, Ahuja T, Petrilli C, Rajagopalan H, Jones S, Rahimian J. Zinc sulfate in combination with a zinc ionophore may improve outcomes in hospitalized COVID-19 patients. J Med Microbiol. 2020;69:1228–1234.
5. Jothimani D, Kailasam E, Danielraj S, Nallathambi B, Ramachandran H, Sekar P, et al. COVID-19: Poor outcomes in patients with Zinc deficiency. Int J Infect Dis. 2020.
